# Th22 is the effector cell of thymosin β15-induced hair regeneration in mice

**DOI:** 10.1186/s41232-023-00316-z

**Published:** 2024-01-08

**Authors:** Nana Tao, Yuyuan Ying, Xie Xu, Qingru Sun, Yaoying Shu, Shiyu Hu, Zhaohuan Lou, Jianli Gao

**Affiliations:** 1 School of Pharmaceutical Sciences, Zhejiang Chinese Medical University, Hangzhou, Zhejiang 310053 People’s Republic of China; 2 The First Affiliated Hospital of Zhejiang Chinese Medical University (Zhejiang Provincial Hospital of Chinese Medicine), Zhejiang Chinese Medical University, Hangzhou, Zhejiang 310053 People’s Republic of China

**Keywords:** Thymosin beta 15, T helper 22, IL-22, Hair follicle, Immune homeostasis

## Abstract

**Background:**

Thymosin beta family has a significant role in promoting hair regeneration, but which type of T cells play a key role in this process has not been deeply studied. This research aimed to find out the subtypes of T cell that play key role in hair regeneration mediated by thymosin beta 15 (Tβ15).

**Methods:**

Ready-to-use adenovirus expressing mouse Tmsb15b (thymosin beta 15 overexpression, Tβ15 OX) and lentivirus-Tβ15 short hairpin RNA (Tβ15 sh) were used to evaluate the role of Tβ15 in hair regeneration and development. The effect of Th22 cells on hair regeneration was further studied by optimized Th22-skewing condition medium and IL-22 binding protein (IL-22BP, an endogenous antagonist of IL-22, also known as IL-22RA2) in both ex vivo culture C57BL/6J mouse skin and BALB/c nude mice transplanted with thymus organoid model.

**Results:**

The results show that Tβ15, the homologous of Tβ4, can promote hair regeneration by increasing the proliferation activity of hair follicle cells. In addition, high-level expression of Tβ15 can not only increase the number of Th22 cells around hair follicles but also accelerate the transformation of hair follicles to maturity. Consistent with the expected results, when the IL-22BP inhibitor was used to interfere with Th22, the process of hair regeneration was blocked.

**Conclusions:**

In conclusion, Th22 is the key effector cell of Tβ15 inducing hair regeneration. Both Tβ15 and Th22 may be the potential drug targets for hair regeneration.

**Supplementary Information:**

The online version contains supplementary material available at 10.1186/s41232-023-00316-z.

## Introduction

Hair plays an important role in the stability of skin tissue, including preventing ultraviolet radiation, regulating body temperature, and releasing secretions [1]. Hair has the capacity to regenerate by the appendage of the skin-hair follicle, in order to retain these functions [2–4]. At present, drugs for treating alopecia mainly play a role by reducing hair follicle loss or promoting hair growth, but hair transplantation only transfers hair follicles from healthy parts to damaged parts, which cannot solve the root of the problem. Therefore, encouraging the regeneration of hair follicles has emerged as the solution to the issue of hair loss. Studies on hair follicle repair conducted domestically and abroad reveal that the immune system and lymphatic system derived from skin seed cells from the epidermis and dermis are the key variables impacting hair follicle reconstruction. It has been demonstrated that the skin immunological microenvironment is crucial for encouraging hair regeneration [5].

Thymus, the important lymphatic organ, plays a vital role in the development and function of immune system [6, 7]. It is also closely related to hair growth. Recent studies have shown that thymosin beta 4 (Tβ4), a member of thymosin beta family, plays a certain role in hair growth [8]. For example, Tβ4 can promote the proliferation and differentiation of hair follicle stem cells and increase the proportion of hair growth cycle, thus promoting hair regeneration [8, 9]. In addition, Tβ4 can inhibit excessive inflammatory reaction and help maintain a healthy environment for hair follicles. As another member of thymosin beta family, Tβ15 is the thymosin with the highest affinity to G-actin (cytoplasmic single polypeptide chain in non-muscle cells, essential to maintain cell structure, cell adhesion and migration). Its main functions include promoting cell proliferation, reducing inflammatory reactions, and promoting wound healing [10]. Tβ15 also plays a key role in the decreased output of mature T cells [10]. Although there are no research results about Tβ15 in hair regeneration, considering its biological functions of promoting stem cell proliferation and differentiation, anti-inflammatory and anti-oxidation, and the activity of thymosin in promoting hair growth, we speculate that Tβ15 may promote hair regeneration.

Thymosin plays a key role in promoting the differentiation of T cells into T cell subtypes [11, 12]. These T cell subtypes include regulatory T cells (Treg) and helper T cells (Th). In the aspect of T cell-mediated hair regeneration, the current research mainly focuses on Treg, Th1, and Th17 cells. Treg is a special subgroup of T cells, which main function is to maintain immune balance and prevent excessive immune response, so as not to harm their own tissues [13]. Treg cells localize to the hair follicle niche in the steady-state. It plays a key role in maintaining local immune homeostasis and protecting hair follicles from immune attacks during hair growth, which is helpful to maintain hair growth cycle and reduce hair loss [14]. The number and activation of Treg in skin are closely related to the specific stage of hair follicle cycle [15]. In the absence of skin injury, Treg cell expression of the Notch ligand, Jagged-1 promotes hair follicle stem cell proliferation and differentiation during hair generation [16]. In addition, Treg cells can produce TGF-β, which activated Smad2/3 in hair follicle stem cells and promoted hair follicle stem cell activation and proliferation [14]. Th1 cells and Th17 cells are the other two T cell subsets, which play an important role in skin inflammatory reaction and are related to the growth, development, and regeneration of hair follicles to some extent [17]. Th17 cells may be the initiator of the damage of the hair follicle. But Th17 cells are not cytotoxic enough by themselves to undermine the hair follicle under normal circumstances; CD8^+^ T cells or more powerful Th1 cells are required as followers. Th17 cells can secrete cytokines such as interleukin-17 (IL-17). The Th17/Th1 axis might convert into a Th1-dominant immune status using IL-17 inhibitors, which may lead to the destruction of hair follicles and even hair loss [18]. Although some studies have paid attention to the role of Tβs in hair regeneration, so far, the specific T cell subsets that play a major role in hair regeneration induced by Tβs have not been fully clarified.

Therefore, this study intends to systematically analyze the distribution of T cell subsets in hair follicles treated with Tβ15 and clarify the T cell subsets that play a major role in hair regeneration induced by Tβs. In the current study, we found that the expression level of Tβ15 is closely related to the expression level of CCR10 surface markers of Th22 cells in the skin. This shows that Tβ15 plays an important role in regulating the activity and function of Th22 cells and plays a key role in guiding the activation of hair follicle stem cells and the process of hair regeneration. Our research shows that a high level of Tβ15 expression can not only increase the number of Th22 cells around hair follicles but also accelerate the transformation of hair follicles to maturity. It may be that Tβ15 upregulates the expression of IL-6 and other cytokines, induces the differentiation and development of Th22 cells, and thus promotes the activation and proliferation of hair follicle stem cells. These data identify Tβ15 between Th22 cells and hair follicle stem cells as essential for hair regeneration.

## Methods and materials

### Animal model

All the mice were purchased from SIPPR-BK Corporation (Shanghai, China), and all animal protocols were approved by the Institutional Animal Care and Use Committee at Zhejiang Chinese Medical University Laboratory Animal Research Center (Permit Number: SYXK (Zhejiang, China) 2021-0012) and were performed in accordance with the relevant institutional and national guidelines and regulations. Suckling C57BL/6J mice (male, 6–8 weeks old) were used for the back skin organ culture [19]. C57BL/6J mice (male, 1–2 weeks old) were used for the thymus organ culture, and BALB/c nude mice (female, 4–6 weeks old) were used in organoid transplant experiments.

A mice model of organoid transplantation was established according to our published protocol [10].

### Cell infection, transfection, and screening

TECs is one of the main cell types in the basic research of immunology, which can regulate the positive and negative selection of T lymphocytes by secreting different kinds of cytokines. And it also has the function of secreting thymosins, which can support the hair growth of skin tissue ex vivo [21–24]. Immortalized mice thymic epithelial cells (iTECs) were constructed according to our published protocol [20].

For all adenovirus infections, polystyrene (40 μg/mL, Sigma Aldrich, St. Louis, MO, USA) was added to the culture medium to improve the infection efficiency. For lentiviral infection, a selection of puromycin-resistant iTECs was carried out 48 h after transfection by the addition of 3 μg/mL puromycin (Sangon Biotechnology, Shanghai, China). Transfection efficiency was measured using qRT-PCR. All information is provided in Supplementary Fig. 1 and Table 1.

**Table 1:** Sequences of primer pairs for quantitative Real-Time PCR

Gene name	Forward primers (5′-3′)	Reverse primers (5′-3′)
*GAPDH*	GGCTGCCCAGAACATCAT	CGGACACATTGGGGGTAG
*Tmsb15*	CGGCAGACAAGATGAGCGATA	ACCTTTGCAGCCAGGGTAGTA
*Tnfa*	TACAGCGACACTTGACACCC	TGCGGACCATAGAGAGTGGA
*Il6*	TGAACTCCTTCTCCACAAGCG	GCCTCTTTGCTGCTTTCACA
*Il1b*	TGCCACCTTTTGACAGTGATG	GTGCTGCTGCGAGATTTGAA
*Tgfb*	ATCTCGATTTTTACCCTGGTGGT	CTCCCAAGGAAAGGTAGGTGATAGT

*Abbreviations*: *GAPDH* glyceraldehyde 3-phosphate dehydrogenase, *Tmsb15* thymosin beta 15, *Tnfa* tumor necrosis factor alpha, *Il6* interleukin 6, *Il1b* interleukin 1 beta, and *Tgfb* transforming growth factor beta

### Ex vivo mice skin organ culture system

The back skin of mice was extracted after depilation. Remove subcutaneous fat from the skin using forceps and wash with PBS containing 1 × concentration of penicillin-streptomycin. Prepare 24-well culture plates with 0.5 mL of media with 1 cm × 1 cm surgical gelatin sponge. Allow foam to absorb media. The culture media should completely saturate the surgical sponge and fill the well by approximately 25%. Cut tissue into 0.5 cm × 0.5 cm squares and place the skin, dermis side-down, on gelatin sponge squares pre-saturated with culture media. Ensure that the epidermis maintains an air interface and that the dermis is in contact with the media-soaked sponge. The tissue should not be submerged in media. Incubate tissue at 37 °C and 5% CO_2_. Change media every 2–3 days by aspirating media from the well using a 1-mL sterile serological pipette. Among them, except for the control group, the other groups need to lay the iTECs under the sponge block in advance for iTECs secreting thymosins and supporting the hair growth of skin tissue. Therefore, we take iTECs cells as a cell model, set up the iTECs group, and observe the regulatory effect of Tβ15 on hair growth and differentiation and development of Th22 cells at normal level. As described in the “Cell infection, transfection, and screening” section, we used virus transfection technology to selectively overexpress/knock down the Tβ15 gene in iTECs cells and set up the Tβ15 OX/sh group. At the same time, set up a virus control group (pAD-Amp-ox group and pLent-U6-GFP-Puro-sh group). The differentiation-promoting growth factors of the Th22-SCM group are as follows: IL-1β (211-11B, peprotech, 10 ng/mL), IL-6 (216-16, peprotech, 30 ng/mL), IL-23 (200-23, peprotech, 20 ng/mL), FICZ (GC36043, glpbio, 400 nM), galunisertib (LY2157299, glpbio, 10 μM) [25]. The IL-22BP group was cultured with 1 μg/mL IL-22 antibody (16-7222-82, Thermo Fisher Scientific, Waltham, MA, USA) [26]. After being cultured ex vivo for 7 days, all skin organs were used in subsequent experiments [19].

### Ex vivo mice thymus organ culture system

The thymus of the mice was extracted and isolated. Tissue was cultured at 37 °C and 5% CO_2_. The medium was aspirated from the well by using a 1 mL sterile serum pipette. Among them, except the control group and Th22-SCM group, the other groups were intervened with corresponding cell supernatants. The culture conditions of the Th22-SCM group are the same as above. After being cultured ex vivo for 36 h, all of the thymus organs were used in subsequent experiments.

### Hematoxylin and eosin (H&E) staining

Tissue samples were fixed in 10% formalin for 48 h, then dehydrated in gradient ethanol (50%, 75%, 85%, 95%, 100%), transparent with xylene, embedded in paraffin, and sliced at 4 μm. Paraffin sections were hydrated after xylene and gradient ethanol treatment and stained with hematoxylin for 10 min and eosin for 30 s. Images were captured with MoticAE 2000 microscope and quantified using Image J (2.3.0).

### Immunohistochemistry

After dewaxing and antigen repair (citrate antigen repair buffer), paraffin sections were sealed with 3% hydrogen peroxide for 15 min. The primary antibodies for CD4 (13573, Santa Cruz; 1:150), CD8 (18913, Santa Cruz; 1:150), CCR10 (22071-1-AP, proteintech; 1:150), CXCR-3 (137140, Santa Cruz; 1:150), CCR4 (YT5290, Immunoway; 1:150), CCR6 (ab273580, abcam; 1:150), and Foxp3 (53876, Santa Cruz; 1:150) were incubated overnight at 4 °C. Then, sections were incubated with a rabbit anti-mouse enhanced polymer antibody (pv-9000, Biosharp, Hefei, China), and reactions were developed in diaminobenzidine and nuclei counterstained with hematoxylin. Images were captured with a MoticAE 2000 microscope and quantified using Image J (2.3.0).

### Immunofluorescence staining

Formalin-fixed and paraffin-embedded tissues were sliced at 4 μm. Tissue sections were placed in sodium citrate antigen repair buffer. The processed sections were stained with primary antibodies as follows: mouse monoclonal PCNA (56, Santa Cruz; 1:150) antibody, rabbit anti-mouse CCR10 (22071-1-AP, proteintech; 1:150) antibody, and mouse monoclonal Tβ15(271649, Santa Cruz; 1:150) antibody. The secondary antibodies are as follows: mouse anti-rabbit IgG-FITC (K2017, Santa Cruz, CA, USA; 1:200); F (ab’) 2-goat anti-mouse IgG (H+L) Alexa Fluor 594 (GAR48810900223, Thermo Fisher Scientific, Waltham, MA, USA; 1:200); and the blue fluorescence was a DAPI-labeled nucleus (Sigma-Aldrich, St. Louis, MO, USA). Pictures were taken with a digital pathological section scanner (OLYMPUS, VS120-S6-W, Tokyo, Japan).

### RNA isolation, reverse transcription, and quantitative Real-Time PCR

The total RNA was extracted from the cells by an RNA-Quick Purification Kit. Then, cDNA was synthesized using an M-MuLV First Strand cDNA Synthesis Kit, and real-time PCR was performed using SYBR Green PCR Mix. All reagents were from Sangon (Shanghai, China). The relative quantification of the genes was performed with the 2^−ΔΔCt^ method, and gene expression was normalized to *GAPDH*. The primers used in this study are listed in Table 1.

### Flow cytometry

The organs of thymus were dissected and smashed through a 70-μm strainer (Thermo Fisher Scientific, Waltham, MA, USA). The distribution of the number of Th22 cells subsets in the thymus was studied with three-color staining performed on freshly isolated thymocytes using the following antibodies: CD3 FITC (553061, BD Pharmingen, San Diego, CA, USA), CD4 APC (553051, BD Pharmingen, San Diego, CA, USA), CCR10 (22071-1-AP, proteintech; 1:150), and fluorescent secondary antibody PE Conjugated Goat anti-Rabbit IgG (H+L) (P-2771MP, Thermo Fisher Scientific, Waltham, MA, USA; 1:100). All flow cytometry data were collected on a flow cytometer (CytoFLEX Beckman, California, USA) and analyzed by using the CytExpert 2.4 software.

### Data analysis

Unless otherwise specified, all quantitative results are expressed as SEM ± average. All statistical analyses were analyzed by Prism 8.0 (GraphPad software). *p* < 0.05 is considered significant.

## Results

### Tβ15 can promote hair growth of mice both ex vivo and in vivo

**Fig. 1:**
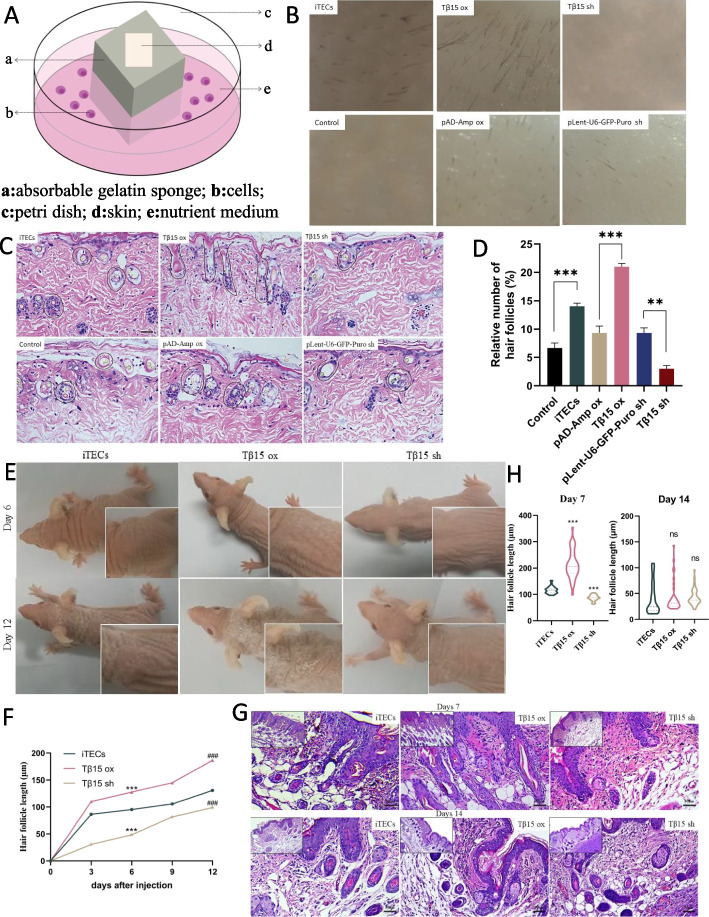
Tβ15 can promote hair growth of mice both ex vivo and in vivo. **a** Simulation diagram of skin organ culture ex vivo. **b** The skin of C57 mice aged 6–8 weeks was cultured ex vivo for 7 days. **c** The representative images of skin cultured ex vivo for 7 days were stained by H&E. Scale bar = 50 μm. **d** Quantitative estimation of relative number of hair follicles in H&E stained images. Observed and counted the number of hair follicles in six different areas of the skin with a magnification of × 100 under an optical microscope (*n* = 3). **e** The nude mice transplanted with organoid organs in both armpits were observed and recorded for 12 days (*n* = 3). **f** From the injection of organs, the back hair of mice was measured and recorded every 3 days (*n* = 18). **g** H&E staining was performed on the skin of the organoid transplant site in mice; scale bar = 100 μm, 50 μm. **h** Quantitative estimation of hair follicle length and measurement with Image J (2.3.0) (*n* = 3). Values are expressed as mean ± SEM. Student’s *t*-test (*p < 0.05, **p < 0.005, ***p < 0.0005)

Tβ4 has been proved to play a key role in the growth and development of hair follicles [8]. As a homologous thymosin of Tβ4, Tβ15 shows great similarity in structure and function [10]. However, few studies have evaluated the role of Tβ15 in the growth and development of hair follicle. In order to verify the promoting effect of Tβ15 on hair growth, a three-dimensional ex vivo culture model of skin organs was established with medical gelatin sponge as scaffold (Fig. 1A). After depilating the back skin of the C57BL/6J mice (6–8 weeks old), it was co-cultured with iTECs (normal; knocked down Tβ15; or overexpressed Tβ15) for 7 days. Results showed that the hair length on the surface of isolated skin organs in the Tβ15 OX group was longer, and the hair growth density was higher. The group of iTECs followed. There was no obvious hair growth on the skin surface of the control group (cultured in medium only) and the Tβ15 sh group (Fig. 1B). The H&E results also showed that there were more hair follicles in the Tβ15 OX group, and the hair follicle cells were larger and more mature (*p* < 0.01) (Fig. 1C, D).

In addition, we established a nude mice model of thymic organ transplantation, which further verified the promoting effect of Tβ15 on hair growth [10]. The results show that Tβ15 can significantly enhance the hair growth (Fig. 1E, F). In addition, Tβ15 significantly increased the induction of growth period and the relative number of hair follicles. Most hair follicle in the Tβ15 OX group broke through the epidermis and extended down to the dermal fat layer, with relatively mature hair follicle cell morphology. In the iTECs group, a small number of hair follicles also broke through the epidermis, but most hair follicles were still in the early stage of growth. At the same time, the number of hair follicles in the normal group and Tβ15sh group was less (Fig. 1G, H). This suggests that Tβ15 can effectively promote the growth of hair follicle ex vivo or in vivo.

### Tβ15 changes the composition of local T cell subsets in hair follicles

**Fig. 2:**
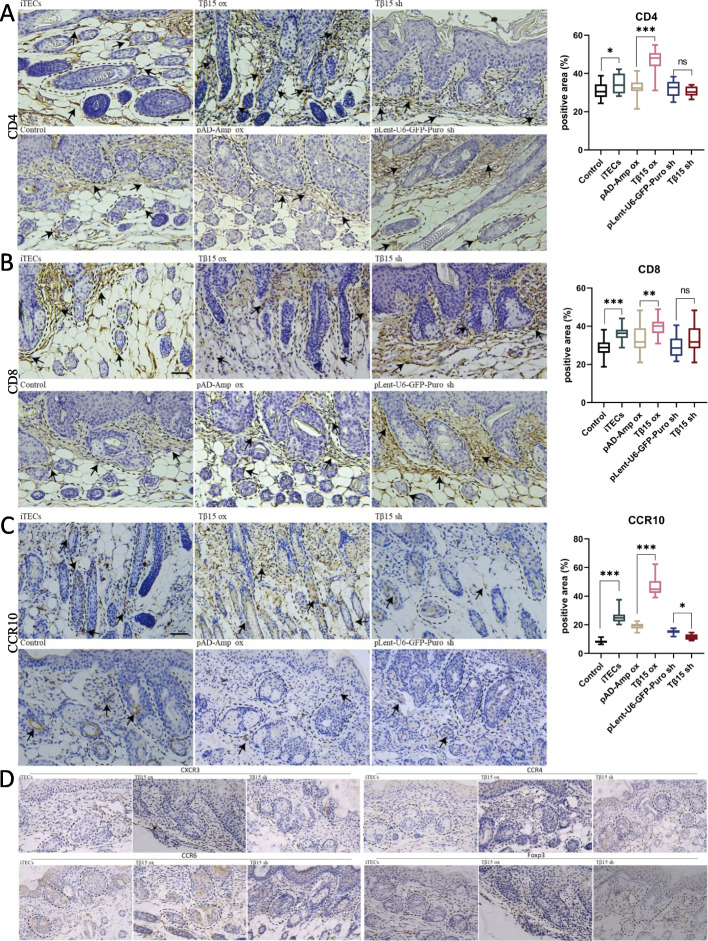
Tβ15 changes the composition of local T cell subsets in hair follicles. **a** The IHC representation of CD4^+^T expression in the skin of the organoid transplantation site of mice on the 7th day was shown; scale bar = 50 μm. **b** The IHC representation of CD8^+^T expression in the skin of the organoid transplantation site of mice on the 7th day was shown; scale bar = 50 μm. **c** The IHC representation of CCR10 expression in the skin of the organoid transplantation site of mice on the 7th day was shown; scale bar = 50 μm. **d** The IHC representation of CXCR3, CCR4, CCR6, and Foxp3 expression in the skin of the organoid transplantation site of mice on the 7th day was shown; scale bar = 50 μm. The data are expressed as the percentage of the expression area of related proteins around hair follicles (per 100 μm^2^ area). Each symbol represents a single data, and all data come from at least three independent experiments. Values are expressed as mean ± SEM. Student’s *t*-test (*p < 0.05, **p < 0.005, ***p < 0.0005)

Considering that β thymosin plays a key regulatory role in the immune system, we detected the expression of CD4^+^T cells and CD8^+^T cells in the local immune microenvironment of skin hair follicles. The results showed that the expression of CD4^+^T cells around hair follicles in the Tβ15 OX group was significantly higher than that in other groups, but there was no significant difference between CD8^+^T cells (Fig. 2A, B). In order to further clarify which types of CD4^+^T cells play a major role, we verified the expression of cell markers CCR10 (Th22), CXCR3 (Th1), CCR4 (Th2), CCR6 (Th17), and Foxp3 (Treg) [27, 28]. The results showed that there were a lot of CCR10 positive cells infiltrating around the hair follicle and inside the hair root sheath in the Tβ15 OX group. A small amount of CCR10 positive cells infiltrated around the hair follicle in the iTECs group. However, in the Tβ15sh group, there was little infiltration of CCR10 positive cells either around the hair follicle or inside the hair root sheath (Fig. 2C, D). The above results show that Tβ15 can affect the immune microenvironment around hair follicles, thus promoting hair growth.

### The differentiation-promoting factor of Th22 can promote hair growth

**Fig. 3:**
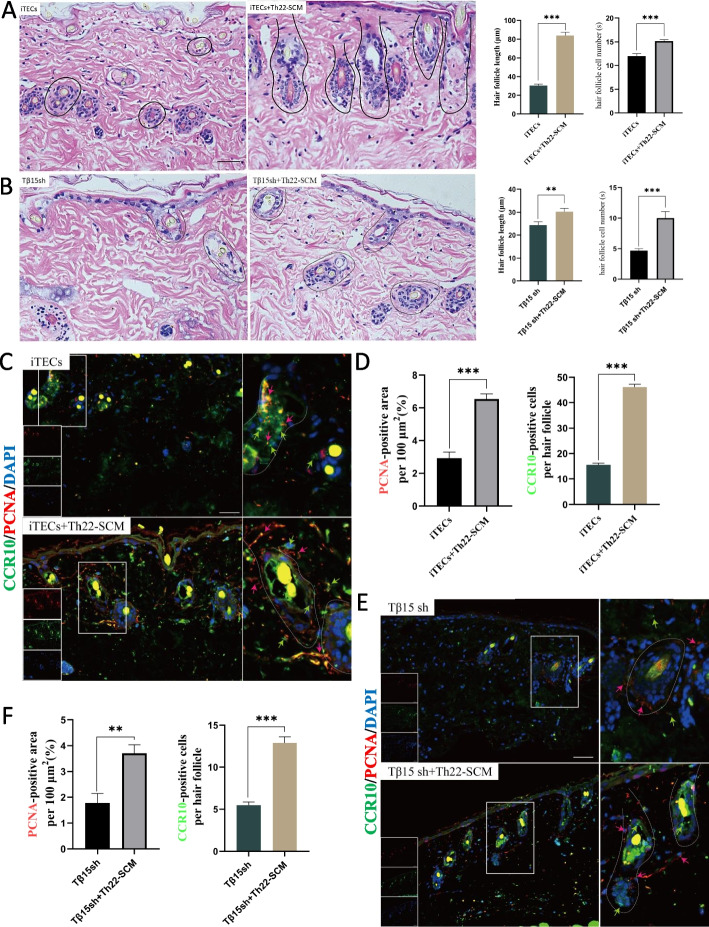
The differentiation-promoting factor of Th22 can promote hair growth. **a**, **b** The representative images of skin cultured ex vivo for 7 days were stained by H&E. Scale bar =50 μm; quantitative estimation of hair follicle length and measurement with Image J (2.3.0) (*n* = 3). **c**, **f** PCNA (red arrow) and CCR10 (green arrow) were stained by immunofluorescence in skin cultured ex vivo for 7 days. The image in the lower right corner enlarges some hair follicles; quantify the fluorescence signal intensity of PCNA at 100 μm^2^ around the hair follicle (*n* = 10); quantify the number of CCR10 positive cells around each hair follicle (*n* = 10). In this process, we avoid selecting the hair shaft with yellow spontaneous fluorescence into the statistical area, that is, only the position of per 100 μm^2^ around the hair follicle is selected, and the expression of related proteins in this range is counted. Values are expressed as mean ± SEM. Student’s *t*-test (*p < 0.05, **p < 0.005, ***p < 0.0005)

The differentiation of T cells is primed by direct contact or the interaction with cytokines produced by APCs and tissue cells and depends on complex lineage-defining transcriptional networks [29]. Recently, the optimized Th22-skewing condition proposed by Plank was widely accepted, which was composed of IL-23, IL-1β, IL-6, and the endogenous AHR ligand 6-formylindolo[3,2-b] carbazole (FICZ) [25]. To verify whether Th22 cells play an important role in hair growth, we used differentiation-inducing factors (IL-23, IL-6, IL-1β, etc.) to carry out intervention culture (Th22-skewing condition medium, Th22-SCM) on isolated skin organs. Compared with the iTECs group, the morphology of hair follicles in the iTECs+Th22-SCM group is more mature (Fig. 3A). Compared with the Tβ15sh group, the number of hair follicle cells and hair follicle nuclei increased relatively after the intervention of Th22-SCM (Fig. 3B). However, compared with the iTECs group, the knock-down of Tβ15 will hinder Th22-SCM from promoting hair growth to some extent. In addition, the results of immunofluorescence showed that the expression of PCNA and CCR10 increased significantly in the presence of Th22-SCM intervention (Fig. 3C–F). Overall, our results provide evidence that Th22 cells are the key cells to promote hair regeneration, but the knock-down of Tβ15 will weaken the role of Th22-SCM in promoting hair growth to some extent.

### IL-22BP blocks Tβ15-induced hair growth in mice

**Fig. 4:**
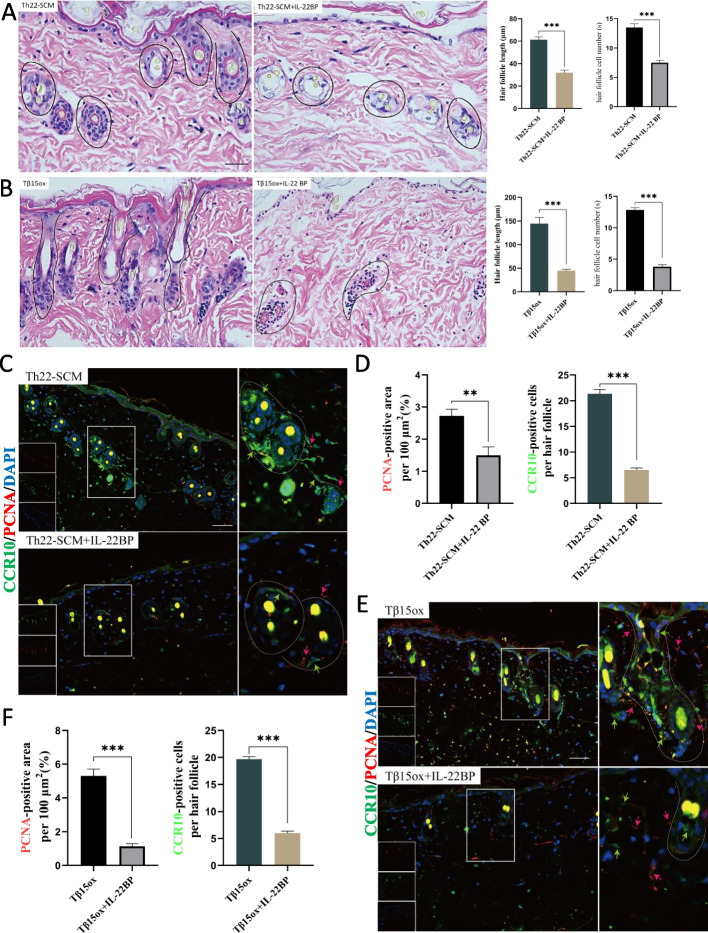
IL-22BP blocks Tβ15-induced hair growth in mice. **a**, **b** The representative images of skin cultured ex vivo for 7 days were stained by H&E. Scale bar = 50 μm; quantitative estimation of hair follicle length and measurement with Image J (2.3.0) (*n* = 3). **c**, **f** PCNA (red arrow) and CCR10 (green arrow) were stained by immunofluorescence in skin cultured ex vivo for 7 days. The image in the lower right corner enlarges some hair follicles; quantify the fluorescence signal intensity of PCNA at 100 μm^2^ around the hair follicle (*n* = 10); quantify the number of CCR10 positive cells around each hair follicle (*n* = 10). Values are expressed as mean ± SEM. Student’s *t*-test (*p < 0.05, **p < 0.005, ***p < 0.0005)

The main secretion of Th22 is IL-22 [30]. Moreover, studies have shown that IL-22 can promote the proliferation of keratinocytes, inhibit their differentiation, promote the expression of related keratin, control hair follicle circulation, and promote hair growth [31–33]. Therefore, IL-22BP was used as Th22 inhibitor and co-cultured with mice skin ex vivo. Compared with the Th22-SCM group, when IL-22BP inhibitor was added for joint intervention, serious vacuolation of hair follicles occurred. Moreover, the hair follicle cells in the Th22-SCM+IL-22BP group were smaller in morphology and fewer in nucleus (Fig. 4A). Compared with the Tβ15 OX group, when IL-22BP inhibitor was added for joint intervention, the normal regeneration of hair follicles was significantly inhibited (Fig. 4B). At the same time, immunofluorescence results showed that the number of proliferative cells around hair follicles and the number of Th22 cells decreased significantly in the presence of IL-22BP inhibitor (Fig. 4C–F). In a word, our results show that the endogenous inhibitor IL-22BP can inhibit the proliferation and normal development of hair follicle cells. At the same time, IL-22BP can also block the effects of Th22 differentiation-promoting factor and Tβ15 and prevent the normal hair follicle regeneration.

### Tβ15 induces the differentiate of mice thymocytes into Th22 cells

**Fig. 5:**
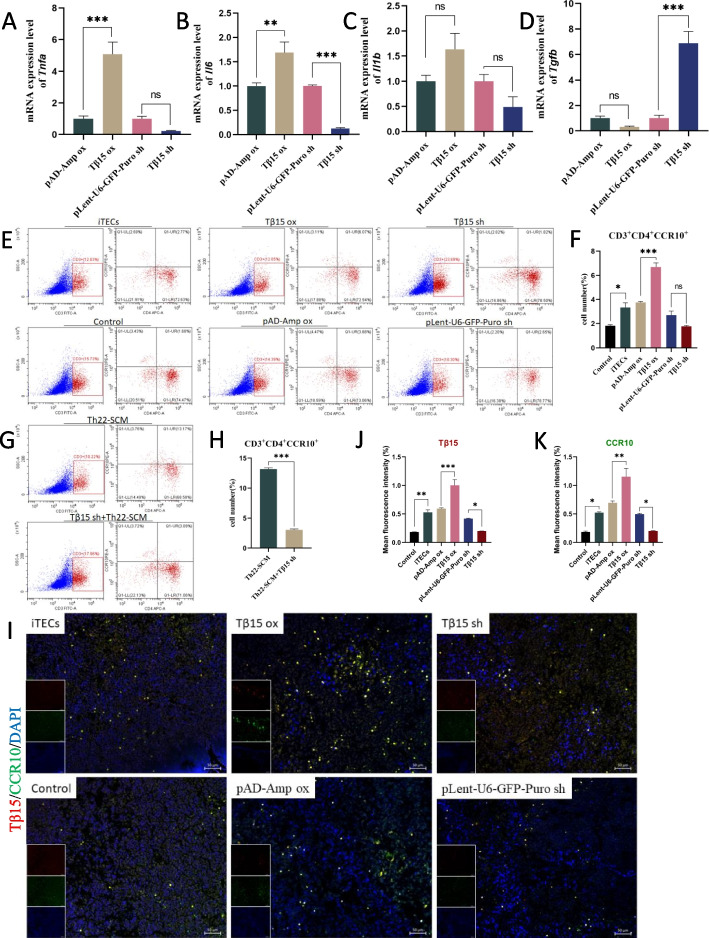
Tβ15 induces the differentiate of mice thymocytes into Th22 cells. **a**, **d** The mRNA levels of *Tnfa*, *Il6*, *Il1b*, and *Tgfb* in different groups of iTECs. For each sample, the corresponding *GAPDH* mRNA level was used to standardize the mRNA level. **e**, **h** Comparison of T cell differentiation in thymus of young rats cultured ex vivo in different groups. Frequencies of Th22 cells (CD3^+^CD4^+^CCR10^+^) are shown as percentage of total cells. **i** Tβ15 and CCR10 were stained with immunofluorescence in the thymus of young rats ex vivo*.* **j**, **k** Statistical diagram of the expression of Tβ15 and CCR10; values are expressed as mean ± SEM. Student’s *t*-test (*p < 0.05, **p < 0.005, ***p < 0.0005)

Studies have shown that IL-6, TNF-α, and other cytokines can induce the differentiation of Th22 cells, while transforming growth factor beta (TGF-β) can inhibit the differentiation of Th22 cells at high concentration [34]. Therefore, the expression levels of *Il6*, *Tnfa*, *Il1b*, and *Tgfb* mRNA in iTECs cells were verified. The results showed that the expressions of *Tnfa* and *Il6* increased relatively after overexpression of Tβ15, while *Tgfb* shows an opposite downward trend (Fig. 5A–D). This indicates that when skin is co-cultured with iTECs, the intervention of Tβ15 may promote the secretion of related cytokines by iTECs cells and promote the changes of T cell subsets in skin tissue. In addition, thymus is the main place for the differentiation and development of T lymphocytes. We intervened the thymus of 7-day-old mice with Tβ15 and Th22 differentiation-promoting factors respectively. The results of flow cytometry showed that the number of CD3^+^CD4^+^CCR10^+^ cells in the Tβ15 OX group was significantly higher than that in the pAD-Amp ox group, and there was a significant difference (7.28% vs. 3.88%, *p* < 0.001, Fig. 5E, F). This indicates that overexpression of Tβ15 will promote the maturation and differentiation of T lymphocytes in the thymus. It was also observed that the number of CD3^+^CD4^+^CCR10^+^ cells in the Th22-SCM intervention group decreased by approximately 4.3-fold after Tβ15 knockdown (*p* < 0.001). This indicates that the knock-down of Tβ15 will weaken the differentiation-promoting effect of Th22 differentiation-promoting factor (Fig. 5G, H). In addition, immunofluorescence results also showed that when Tβ15 was overexpressed, the expression levels of Tβ15 and CCR10 in thymus increased significantly, and the expression positions of Tβ15 and CCR10 were positively correlated (Fig. 5I–K). These results indicate that Tβ15 may promote the secretion of related cytokines, thus changing the composition of T cell subsets around hair follicles, inducing T lymphocytes to differentiate into Th22 cells, and promoting hair regeneration.

## Discussion

At present, the number of patients with alopecia is increasing rapidly, regardless of gender and age [35, 36]. Hair loss is mainly due to the decrease of hair follicle stem cell activity and hair follicle regeneration ability [37]. The existence of hair follicles not only provides skin functional support but also is an important basis for maintaining skin homeostasis and self-renewal [3]. In this study, we found that after the specific overexpression of Tβ15, the speed of hair follicle transformation to growth period as well as the shape and number of hair follicles changed. Further research shows that Tβ15 can regulate the proliferation and regeneration of hair follicles by changing the composition of T cells around hair follicles. Besides, Tβ15 also regulates the differentiation and maturation of T lymphocytes in thymus, especially the differentiation of Th22 cells.

Previous studies have shown that Tβ4 has many potential functions in the growth and development of hair follicles. Endogenous Tβ4 can activate the hair follicle cycle transition in mice and affects hair follicle growth and development by promoting the migration and differentiation of hair follicle stem cells and their progeny [38, 39]. And with the establishment and improvement of cloning technology, the influence of exogenous Tβ4 on the growth and development of hair follicles was also investigated through the generation of a mouse model overexpressing Tβ4. In 2007, Philp et al. generated transgenic mice overexpressing Tβ4 driven by the keratin 5 promoters [39]. When the rate of hair regrowth was measured after shaving the hair of resting 8-week-old mice, hair in Tβ4-overexpressing mice grew faster than that in wild-type mice from the same litter. Such results were also verified in mouse models generated by Cha et al. [40]. In summary, in the last decade of research, different teams have shown that exogenous Tβ4 promotes the growth and development of hair follicles in mice and increases the rate of hair growth [41–46]. In addition, thymosin also plays an important role in the differentiation and development of T cells. Studies show that Tβ4 can effectively balance the activities of Th1 and Th2 cells as well as the concentrations of proinflammatory and anti-inflammatory cytokines to restore normal immune homeostasis [47]. Transactions on Speech and Language Processing (TSLP) promoted B cell proliferation, and TSLP-activated B cells polarized CD4^+^ naive T cells into follicular helper T (Tfh) cells through OX40L [48]. TSLP can also promote the polarization of helper T cells to Th2/Th22 [49, 50]. And thymosin α1 can increase the level of Tregs and the production of inflammatory cytokines TNF-α, IL-1β, and IL-6 [51]. It can also induce the immune response of Th1/Th2 cells and promote the transformation of Th1 cells into Th2 cells [52–55]. However, the importance of Tβ15 in hair follicle regeneration and its functional consequences on T cell differentiation and development have not been fully studied.

The results of this study showed that Tβ15 can accelerate the transformation of hair follicles to maturity and increase the number of hair follicles in the skin organ culture system. However, knocking down Tβ15 will lead to a significant decrease in the number of hair follicles in the skin. After we put the thymic organs into nude mice, we observed that Tβ15 OX can greatly promote the hair growth in nude mice, especially in 2 weeks. Therefore, we speculate that Tβ15 also has a potential role in hair regeneration. In this study, we also observed that the number of CD4^+^T cells and CD8^+^T cells around hair follicles in mice with high level of Tβ15 in iTECs increased significantly. In view of the above situation, we analyzed which type of T cells Tβ15 mainly regulates. The results showed that the high expression of Tβ15 seemed to affect the number of Th22 cells around hair follicles. In contrast, the number of Th22 cells around hair follicles in the Tβ15 knock-down group did not increase. Moreover, the number of other types of T cells, such as Th1 and Th17, did not change with the high expression or knock-down of Tβ15.

Th22 cells, as a newly defined CD4^+^T cell lineage, mainly secrete cytokines such as IL-22, which play an important role in skin homeostasis and pathological state [56]. Our results showed that the number of hair follicles in the skin of Th22 differentiation-promoting factor intervention group increased significantly, and the shape of hair follicles was closer to the mature state. At the same time, there are more Th22 cells around the hair follicle. However, this effect will be inhibited with the knocking down of Tβ15. In addition, we proved that IL-22BP can not only inhibit the effect of Th22 differentiation-promoting factor but also prevent the normal hair follicle regeneration under the condition of Tβ15 OX. In addition, cytokines such as IL-6 are the influencing factors of T cell differentiation into Th22 cells. We found that these cytokines are regulated by Tβ15. *Il6*, *Il1b*, and *Tnfa* are all expressed at high level in iTECs of Tβ15 OX. On the contrary, *Tgfb* is expressed at a low level in iTECs of Tβ15 OX. At the same time, in in vitro culture of thymus organs, we found that knocking down Tβ15 not only inhibited the differentiation and development of T lymphocytes into Th22 cells but also prevented the function of Th22 differentiation-promoting factor. Our results further support that Th22 cells may be the key cells in promoting hair follicle regeneration by Tβ15. Tβ15 can regulate the differentiation and development of Th22 cells and promote hair regeneration by upregulating the expression of cytokines such as IL-6.

In a word, our research results strongly indicate that Tβ15 is involved in controlling the differentiation and development of T lymphocytes and can regulate the immune homeostasis around hair follicles together with Th22 cells to promote hair regeneration. At the normal level of Tβ15, the normal proliferation and development of hair follicles can be maintained. A high level of Tβ15 will increase the number of Th22 cells around hair follicles and accelerate the transformation of hair follicles to mature state. However, knocking down Tβ15 will inhibit the differentiation of Th22 cells and the growth of hair follicles. Our current results further expand our understanding of the biological functions of Tβ15 and Th22 cells and provide a new insight into the influence of T cells on hair regeneration. It is further suggested that Tβ15 and Th22 may be potential drug targets for hair regeneration. However, there are still some limitations in this study. This study does not involve deeper mechanism research and cannot explain our findings in depth. But this is also the direction we hope to study further in the future, and we plan to add more data in the future.

## Conclusion

Our results emphasize that Th22 is the key effector cell of hair regeneration induced by Tβ15. Tβ15 may induce T lymphocytes in the skin to transform into Th22 subtype by changing the local immune environment of hair follicles, thus promoting hair regeneration. In a word, these findings suggest that Tβ15 and Th22 cells may be potential drug targets to promote hair regeneration.

### Supplementary Information

**Additional file 1:** **Supplementary Figure 1.** The Tβ15 gene overexpression and knocking down efficacy in iTECs lines. (a) Representative images of iTECs-pLent-U6-GFP-Puro shRNA and iTECs-Tβ15 shRNA. Images are taken with respective channels for bright field (BF) and GFP. Scale bar =20 μm. (b) qRT-PCR shows the *Tmsb15* gene overexpression and knocking down efficacy in iTECs. The corresponding *GAPDH* mRNA level was used to standardize the mRNA level. Values are expressed as mean ± SEM. Student’s t-test (*p < 0.05, **p < 0.005, ***p < 0.0005).

## Data Availability

The datasets used and/or analyzed during the current study are available from the corresponding author on reasonable request.

## Abbreviations

Tβ4: Thymosin beta 4
Tβ15: Thymosin beta 15
Th22 cell: T helper 22 cell
Th1 cell: T helper 1 cell
Th2 cell: T helper 2 cell
Th17 cell: T helper 17 cell
Treg: Regulatory T cell
IL-1β: Interleukin-1 beta
IL-6: Interleukin-6
IL-13: Interleukin-13
IL-22: Interleukin-22
IL-23: Interleukin-23
IL-22BP: Interleukin-22 binding protein
TNF-α: Tumor necrosis factor-alpha
TGF-β: Transforming growth factor-beta
qRT-PCR: quantitative Real-Time PCR
PBS: Phosphate-buffered saline
FICZ: 6-Formylindolo [3,2-b] carbazole
AHR: Aryl hydrocarbon receptor
H&E: Hematoxylin and eosin
GAPDH: Glyceraldehyde-3-phosphate dehydrogenase
IHC: Immunohistochemistry
MMP: Mechanism metalloproteinases
TSLP: Transactions on Speech and Language Processing
