# Supplementary figures and images for: Th22 is the effector cell of thymosin β15-induced hair regeneration in mice
Source: Inflamm Regen. 2024 Jan 8;44:3. doi: 10.1186/s41232-023-00316-z (PMC10773137; doi:10.1186/s41232-023-00316-z)

**A**

pLent-U6-GFP-Puro sh

T $\beta$ 15 sh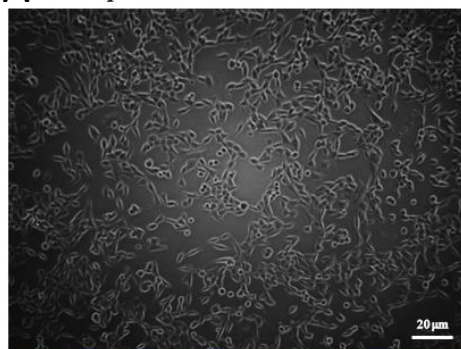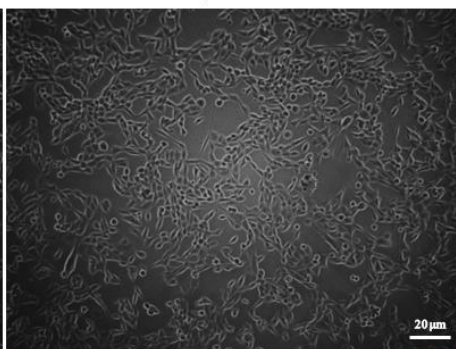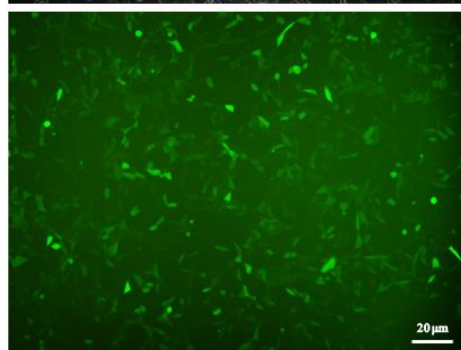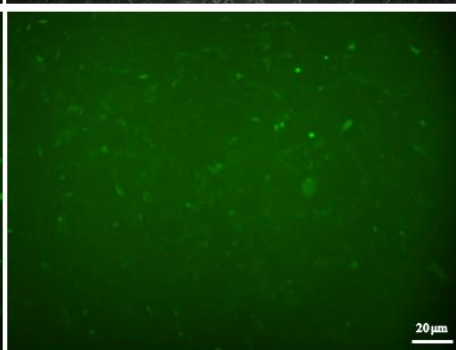**B***Tmsb15*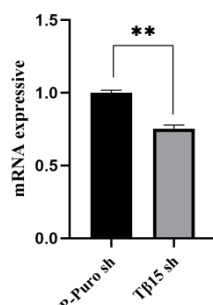*Tmsb15*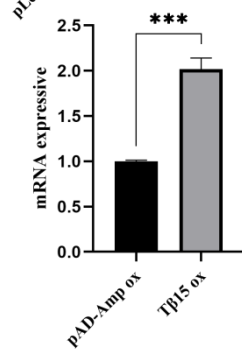

Supplement: Supplementary file 1 — Additional file 1: Supplementary Figure 1. The Tβ15 gene overexpression and knocking down efficacy in iTECs lines. (a) Representative images of iTECs-pLent-U6-GFP-Puro shRNA and iTECs-Tβ15 shRNA. Images are taken with respective channels for bright field (BF) and GFP. Scale bar =20 μm. (b) qRT-PCR shows the Tmsb15 gene overexpression and knocking down efficacy in iTECs. The corresponding GAPDH mRNA level was used to standardize the mRNA level. Values are expressed as mean ± SEM. Student’s t-test (*p < 0.05, **p < 0.005, ***p < 0.0005). [file 41232_2023_316_MOESM1_ESM.pdf]
